# Antigen specific immune response in *Chlamydia muridarum* genital infection is dependent on murine microRNAs-155 and -182

**DOI:** 10.18632/oncotarget.11461

**Published:** 2016-08-20

**Authors:** Rishein Gupta, Tanvi Arkatkar, Jonathon Keck, Gopala Krishna Lanka Koundinya, Kevin Castillo, Sabrina Hobel, James P. Chambers, Jieh-Juen Yu, M. Neal Guentzel, Achim Aigner, Lane K. Christenson, Bernard P. Arulanandam

**Affiliations:** ^1^ South Texas Center for Emerging Infectious Diseases and Center of Excellence in Infection Genomics, University of Texas at San Antonio, San Antonio, TX, USA; ^2^ Rudolf-Boehm-Institute for Pharmacology and Toxicology, Clinical Pharmacology, University of Leipzig, HärtelstraΔe, Leipzig, Germany; ^3^ Department of Molecular and Integrative Physiology, University of Kansas Medical Center, Kansas City, KS, USA

**Keywords:** *Chlamydia muridarum*, host immunity, microRNA-155, microRNA-182, CD4+ T-cells, Immunology and Microbiology Section, Immune response, Immunity

## Abstract

Anti-chlamydial immunity involves efficient presentation of antigens (Ag) to effector cells resulting in Ag-specific immune responses. There is limited information on inherent underlying mechanisms regulating these events. Previous studies from our laboratory have established that select microRNAs (miRs) function as molecular regulators of immunity in *Chlamydia muridarum* (Cm) genital infection. In this report, we investigated immune cell type-specific miRs, *i.e.* miR-155 and -182, and the role in Ag-specific immunity. We observed significant up-regulation of miR-155 in C57BL/6 bone marrow derived dendritic cells (BMDC), and miR-182 in splenic Ag-specific CD4^+^ T-cells. Using mimics and inhibitors, we determined that miR-155 contributed to BMDC activation following Cm infection. Co-cultures of miR-155 over-expressed in BMDC and miR-182 over-expressed in Ag-specific CD4^+^ T-cells, or miR-155^−/−^ BMDC with miR-182 inhibitor treated Ag-specific CD4^+^ T-cells, resulted in IFN-γ production comparable to Ag-specific CD4^+^ T-cells isolated from Cm infected mice. Additionally, miR-182 was significantly up-regulated in intranasally vaccinated mice protected against Cm infection. *In vivo* depletion of miR-182 resulted in reduction in Ag-specific IFN-γ and genital pathology in Cm infected mice. To the best of our knowledge, this is the first study to report an interaction of miR-155 (in Cm infected DC) and miR-182 (in CD4^+^ T-cell) resulting in Ag specific immune responses against genital Cm.

## INTRODUCTION

The anti-*Chlamydia trachomatis* (Ct) immune response involves neutrophils, macrophages, and dendritic cells followed by antigen (Ag)-specific and non-specific T-cells homing to the infected genital tract [1]. Critical interactions of infected mucosal epithelial cells and Ag-specific interferon (IFN)-γ producing CD4^+^ T-cells results in effective anti-Ct immunity [2]. Despite efforts to determine anti-Ct immunity for effective vaccination strategies [3], Ct remains the leading sexually transmitted infection (STI) globally [4], and the most common STI in the US [5]. In infected women, chronic infection or exaggerated immune responses may potentially result in inflammatory pathology in the uterus and fallopian tube, and subsequently pelvic inflammatory disease (PID), and infertility [6]. Given that several laboratories [3, 4, 7-9] including ours [10], have reported that effective anti-Ct vaccination strategies require the targeted induction of adaptive immune responses, focused investigation on the role of underlying molecular modulators that have the ability to regulate Ag-specific immunity is essential and timely.

To this end, we have reported on the role of microRNAs (miRs) as molecular regulators, in the genital tract of *C. muridarum* (Cm, murine strain of genital Ct) infected mice [11]. MicroRNAs are short, non-coding RNA species that post-transcriptionally regulate gene expression by binding to target gene mRNA to decrease translation and increase mRNA degradation [12]. Functionally, miRs have been shown to alter host processes including immunity, inflammation, and reproduction [13-17]. In our initial report, we investigated the contribution of spatio-temporally regulated inflammation and immunopathology associated miRs in anti-Cm immunity in C57BL/6 mice at 6 or 12 days *post* infection [11]. Additionally, we have recently reported the regulation of intracellular adhesion molecule *(ICAM)-1* gene by miR-214 in Cm infected mice [18]. We found that miR-214 regulated *ICAM-1* expression differentially in Cm infected wild type and IL-17A deficient mice and lead to significant differences in upper genital pathology [18] . In addition to our reports on the role of miRs in Cm associated immune response and pathogenesis [11], the growing importance of investigating miRs in Ct infection has been emphasized [19]. Importantly, Igeitseme *et al*., have demonstrated the role of caspase-mediated cleavage inactivation of DICER, the miR biogenesis enzyme, and contribution of selected miRs in mesenchymal-epithelial transition and development of genital pathology in Ct serovar D infected mice [20], [21]. The association of single miR nucleotide polymorphisms and inflammatory genes in Ct infected humans [22], and the potential of miRs as biomarkers for genital Ct has been reported [23].

Anti-Ct immunity *in vivo* results from a complex of various immune cell types including Ag-presenting cells (APC) and CD4+ T-cells [4]. However, the contribution of miRs in initiating or regulating these processes in Ct infection has been not investigated. In the current study, we elucidated the contribution of specific miRs from two immune cell populations, dendritic (DC) and CD4^+^ T-cells (highly effective Ag-presenting cells and the principle effector cells, respectively) involved in anti-Cm immunity [3, 24, 25]. We observed miR-155 and -182 to be significantly up-regulated in Cm infected cultured murine DC, and in Ag-specific murine CD4^+^ T-cells isolated at day 12 *post* infection, respectively. Activation of bone marrow derived DC (BMDC) as assessed by major histocompatibility complex II expression was regulated by miR-155. Co-culture of miR-155 treated BMDC (transfected with a miR-155 inhibitor) or miR-155^−/−^ BMDC with Ag-specific CD4^+^ T-cells resulted in significant up-regulation of IFN-γ production. Ag-specific IFN-γ production was abrogated in total splenocytes from miR-182 inhibitor treated mice compared to scramble treated or mock treated Cm infected mice. Moreover, following Cm infection, miR-182 inhibitor treated mice displayed significant reduction in development of upper genital pathology compared to scramble or mock treated mice. Importantly, IFN-γ production in miR-155 mimic treated BMDC co-cultured with miR-182 mimic treated Ag-specific CD4^+^ T-cells was comparable to untransfected co-cultures. Untransfected co-cultures surrogately demonstrated/ mimicked the specific role of miR-155 and -182 in IFN-γ production during an *in vivo* Cm infection. These findings were further corroborated by comparable IFN-γ production in miR-155^−/−^ BMDC cocultured with CD4^+^ T-cells from miR-182 inhibitor treated mice compared to WT BMDC co-cultured with CD4^+^ T-cells from Cm infected mice. Taken together, these results strongly demonstrate the combined effect of 2 miRs (one up-regulated in the BMDC, and the other in CD4^+^ T-cells) in contributing to anti-Cm immune responses and IFN-γ production reported previously to be critical for protection against a genital Cm infection [1, 4]. To the best of our knowledge, this is the first report describing regulation of IFN-γ production via miR-155 and -182 in Cm infected cells suggesting a role for these miRs in adaptive immunity following infection.

## RESULTS

### Murine miR-155 is significantly up-regulated and contributes to activation of dendritic cells following *Chlamydia muridarum* infection

**Figure 1:**
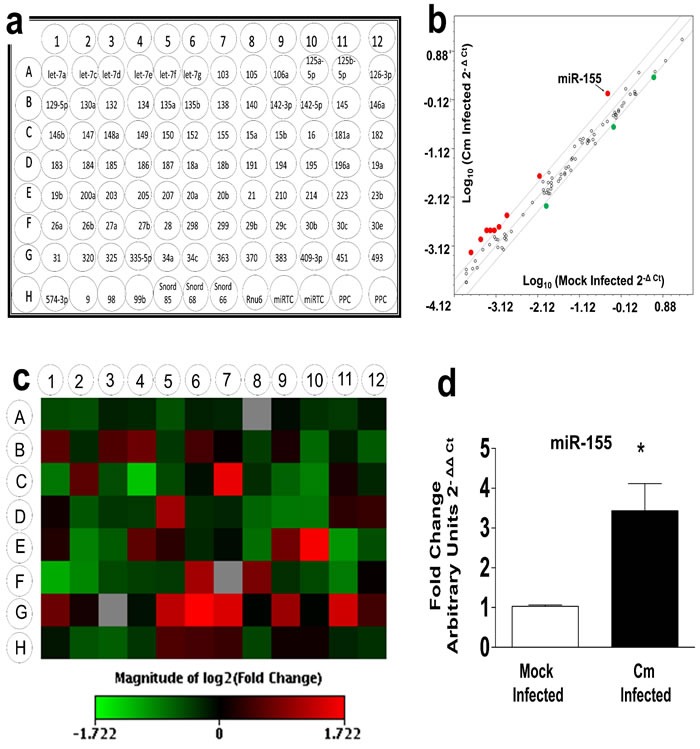
Murine miR-155 is significantly up-regulated in *Chlamydia muridarum* infected dendritic cells Female C57BL/6 mouse bone marrow derived dendritic cells (BMDC) were infected with *Chlamydia muridarum* (Cm, MOI = 1). **a.** ‘Inflammation and Immunopathology’ focused RT2 miRNA plates were procured from Qiagen, Valencia, CA) - **b.** Scatterplot analyses (individual miRs represented by red and green dots as up- or down-regulated respectively (± 2-fold change) and open dots(<± 2-fold change) in Cm infected BMDC compared to mock infected BMDC. **c.** heat map profiles of miRs in RT2 miRNA plates were plotted by comparing miR profiles of Cm infected and mock infected BMDC **d.** MiR-155 specific PCR amplication revealed significant up-regulation compared to mock controls. Results are representative of 3 independent experiments. *P < 0.05 Student's *t* test; black arrow indicates miR-155. Rnu6 and Snord 68 were used as housekeeping miRs. MOI = Multiplicity of infection; miR = microRNA.

Previously, we reported the spatio-temporal regulation of miRs in the genital tract of intravaginally infected C57BL/6 mice at 6 and 12 days *post* Cm infection [11]. We have demonstrated that miR-mediated modulation of host immunity in the genital tract (*i.e.*, miR-214 regulates *ICAM-1*) contributes to upper genital pathology in Cm infected mice [18]. In the current study, we investigated miRs in specific cell types critically needed in generation of anti-Cm immunity [1, 3, 26, 27]. Quantitative reverse transcriptase (qRT) was used to identify miRs in Cm infected BMDC. Using an array spotted with 88 miRs having immune-pathological regulatory function(s) (Figure 1a) [11], we observed 9 miRs up-regulated (red dots Figure 1b), and 3 miRs down-regulated (green dots Figure 1b) to be physiologically regulated (± 2-fold change) in Cm infected BMDC at 24 h *post* infection (Figure 1d, Supplementary Table 1). Importantly, the scatterplot (Figure 1b) and heat map profile (Figure 1c) revealed that the remaining miRs *i.e*., 76 out of 88 (open dots Figure 1b) were regulated in similar fashion to that observed for mock infected BMDC. Therefore, we investigated the role of miR-155 for its well-characterized and extensively reported role(s) in initiating and regulating immune responses [28, 29]. MiR-specific PCR primers confirmed the array results indicating significant up-regulation of miR-155 following Cm infection in BMDC (Figure 1d).

**Figure 2:**
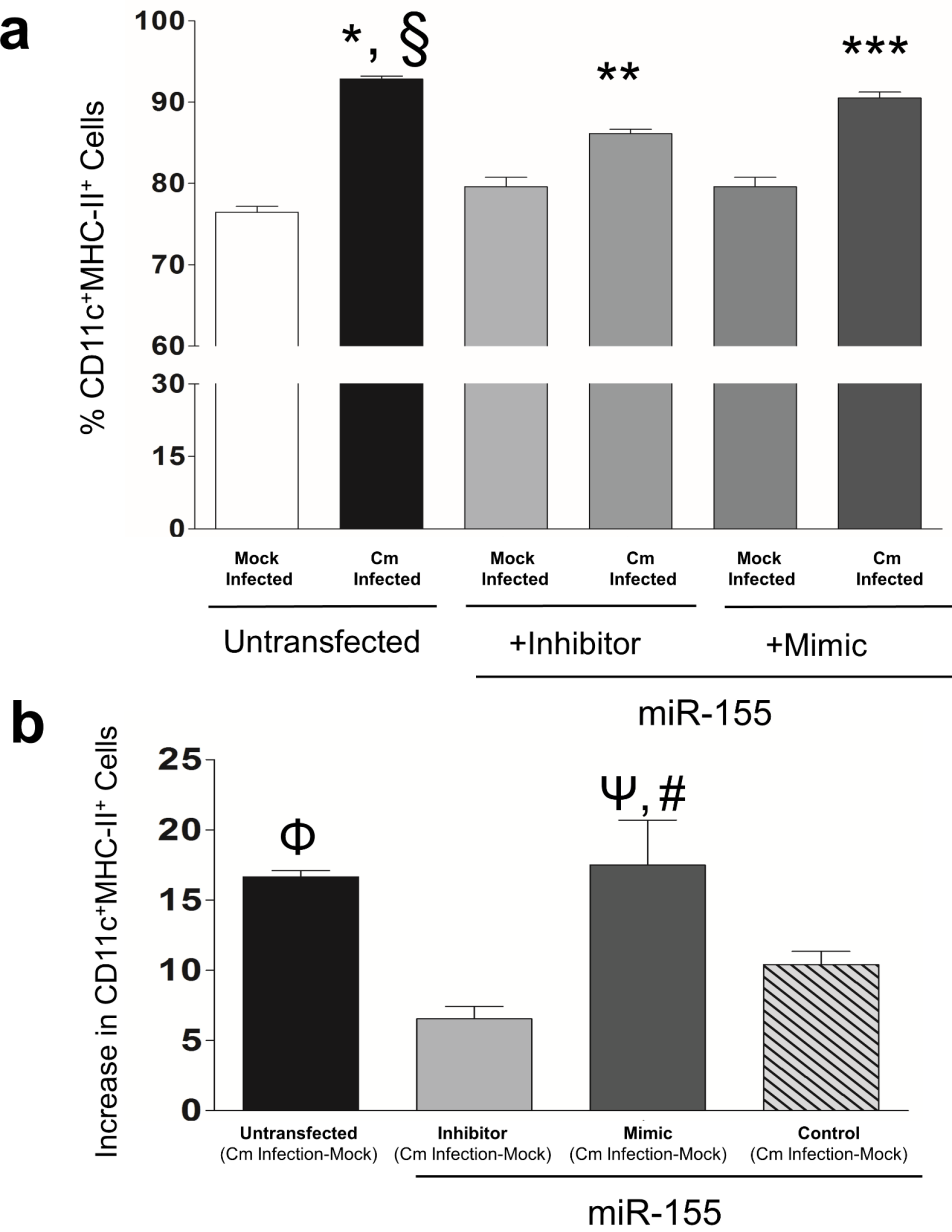
Murine miR-155 regulates expression of Major Histocompatibility Complex II (MHC-II) in *Chlamydia muridarum* infected dendritic cells BMDC were pulsed with Cm (MOI = 1) and 24 hrs later MHC-II expression was determined in CD11c^+^ BMDC using flow cytometry. **a.** Frequency of CD11c^+^MHCII^+^ cells was plotted as % of total cells in Cm or mock infected un-transfected, miR-155 inhibitor or mimic transfected BMDC. Results are representative of 2 independent experiments. *P* < 0.05 using ANOVA with Bonferroni's Multiple Comparison Test.*Cm infected animals compared to mock infected; ^§^Cm infected, untransfected compared to Cm infected, miR-155 inhibitor transfected; **Cm infected compared to mock control infected, miR-155 inhibitor transfected; ***Cm infected miR-155 mimic compared to Cm infected, miR-155 inhibitor transfected BMDC. **b.** Increase in frequency of CD11c^+^MHCII^+^ cells following Cm infection relative to respective mock control infection in untransfected, or miR-155 inhibitor, mimic or control (scramble mimic) was determined. Results are representative of 3 independent experiments. *P* < 0.05 using ANOVA with Bonferroni Multiple Comparison Test*. ^Φ^Cm infected-mock wells in un-transfected wells compared to miR-155 inhibitor wells. ^Ψ^Cm infected mimic compared to miR-155 inhibitor transfected wells. ^#^miR-155 mimic transfected compared to miR-155 control transfected wells.

DCs are professional Ag-presenting cells (APC) that upon activation, initiate adaptive immune responses [26]. MiR-155 regulates DC activation and subsequent T-cell function [30, 31]. Using flow cytometry, we next determined the number of activated cells in miR-155 treated, Cm infected BMDC cultures. A significant increase in the number of CD11c^+^MHC-II^+^ expressing cells (%) in Cm infected BMDC compared to mock infection was observed (Figure 2a). In order to demonstrate ‘cause and effect’ MHC-II^+^ expression was determined in CD11c^+^ BMDC transfected with miR-155 mimics or inhibitors. The percent of CD11c^+^MHC-II^+^ cells was significantly decreased in Cm infected, miR-155 inhibitor transfected BMDC cells compared to Cm infected, untransfected cells (Figure 2a). In contrast, CD11c^+^MHC-II^+^ cells were significantly increased in Cm infected, miR-155 mimic transfected BMDC cells compared to Cm infected, miR-155 inhibitor transfected cells (Figure 2a). Transfection with miR-155 inhibitors that down-regulate endogenous miR-155 in BMDC resulted in a MHC-II^+^ expression change that was significantly reduced compared to miR-155 mimic transfected cells when endogenous miR-155 was overexpressed (Figure 2b). In contrast, BMDC transfected with controls, *i.e.,* scrambled-miRs exhibited significantly lesser MHC-II+ expression change compared to untransfected and mimic transfected cells (Figure 2b). We observed no significant contribution of miR-155 in regulating expression levels of CD40, CD80, and CD86 in Cm infected BMDC (data not shown). Isotype controls for all experimental conditions detected low positivity in CD11c^+^MHC-II^+^ expressing cells (data not shown). Collectively, these results demonstrate that miR-155 contributes to MHC-II expression in CD11c^+^ BMDC following Cm infection.

### Murine miR-155 in *Chlamydia muridarum* infected dendritic cells regulates interferon-γ production

**Figure 3:**
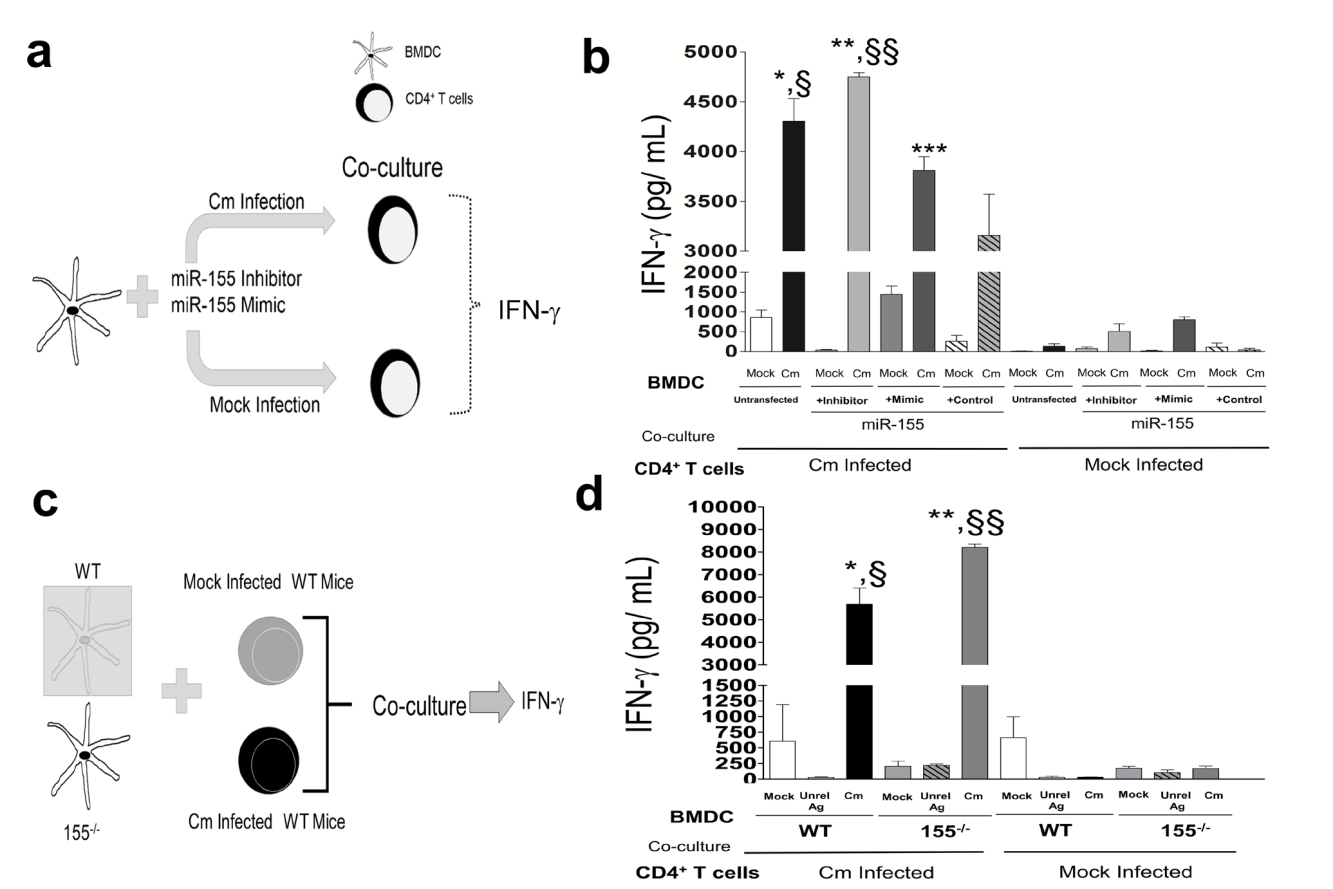
Murine miR-155 in *Chlamydia muridarum* infected dendritic cells regulates interferon-γ production in an antigen specific manner **a.** Diagrammatic representation of BMDC pulsed with Cm (MOI = 1) and co-cultured 24 h later with splenic CD4^+^ T cells isolated at day 12 from mock and Cm infected mice (*n* = 3); **b.** IFN-γ production 72 h *post* co-culture was determined. *P* < 0.05 using ANOVA with Bonferroni's Multiple Comparison Test. *Cm infected, untransfected BMDC compared to mock infected, untransfected BMDC. **Cm infected, miR-155 inhibitor BMDC compared to mock infected control, miR-155 inhibitor BMDC. ***Cm infected, miR-155 mimic BMDC compared to mock infected control, miR-155 mimic BMDC. §Cm infected, untransfected BMDC compared to Cm infected, control transfected (AllStar Negative Control siRNA, Qiagen) BMDC. ^§§^Cm infected, miR-155 inhibitor transfected BMDC compared to Cm infected, miR-155 mimic transfected BMDC. Results are representative of 2 independent experiments. **c.** WT and miR-155^−/−^ BMDC were pulsed with mock, Cm (MOI = 1) or unrelated antigen and co-cultured 24 h later with splenic CD4^+^ T cells isolated at day 12 from mock and Cm infected mice (*n* = 3), **d.** IFN-γ production 72 h *post* co-culture was determined. *P* < 0.05 using ANOVA with Bonferroni's Multiple Comparison Test. Results are representative of 2 independent experiments.

Given the potential of miR-155 regulation of adaptive immunity [28, 32], we investigated the role of miR-155 in mediating Cm-specific immune responses. We co-cultured miR-155 inhibitor or mimic transfected BMDC or miR-155^−/−^ or WT BMDC with splenic CD4^+^ T cells isolated at day 12 from mock or Cm infected mice and measured IFN-γ production by ELISA (Figure 3a, 3c). We observed a significant increase in IFN-γ production in Cm infected BMDC co-cultured with Cm infected CD4^+^T cells, *i.e*., splenic CD4^+^ T cells isolated at day 12 from Cm infected mice compared to mock infected BMDC (Figure 3b). Importantly, a significant increase in IFN-γ production in Cm infected, miR-155 inhibitor transfected BMDC co-cultured with Cm infected CD4^+^ T cells compared to Cm infected, miR-155 mimic transfected BMDC was observed demonstrating a ‘cause and effect’ relationship between miR-155 manipulated DC, and production of IFN-γ when co-cultured with Ag-specific CD4^+^ T cells (Figure 3b). Additionally, the observed interactions between miR-155 in BMDC, and production of IFN-γ was detected specifically upon co-culture with CD4^+^ T cells isolated from Cm infected mice compared to mock infected mice (Figure 3b). We corroborated our results in WT BMDC manipulated with miR-155 mimics and inhibitors (Figure 3a, 3b) by comparing production of IFN-γ in Cm infected WT and miR-155^−/−^ BMDC (Figure 3c, 3d). Similar to responses in Figure 3b, we observed a significant increase in IFN-γ production in Cm infected 155^−/−^ BMDC compared to WT BMDC upon co-culture with Cm infected CD4^+^ T cells (Figure 3d). Taken together, these results demonstrate that miR-155 in BMDC affects Th1 clonal expansion and specifically, IFN-γ production following interactions with Cm-infected CD4+ T cells.

### MiR-182 is significantly regulated in CD4^+^ T cells from *Chlamydia muridarum* infected or vaccinated mice and contributes to Ag-specific immune response in vivo

**Figure 4:**
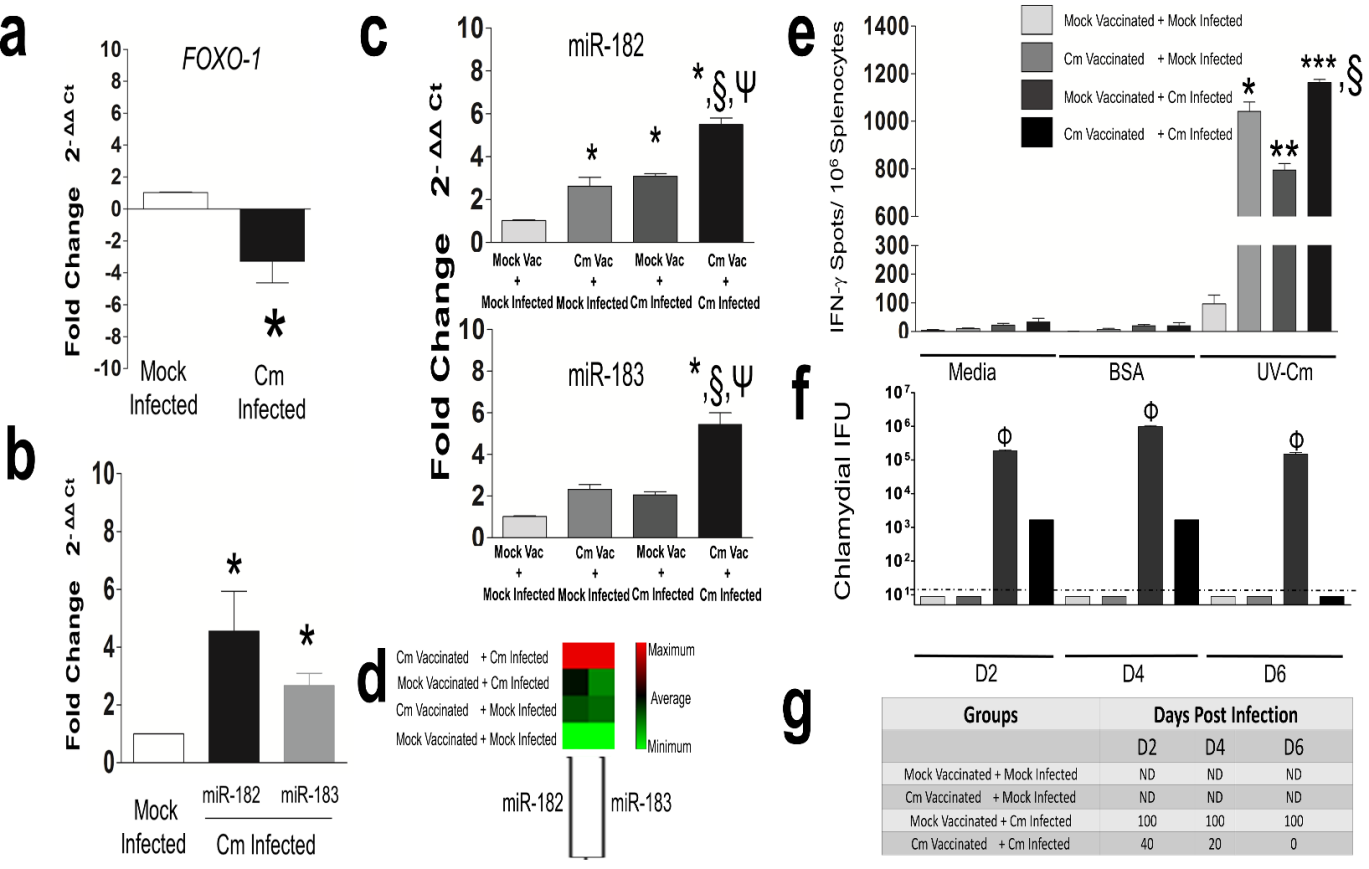
Murine *FOXO-1* and miR-182 is significantly regulated in *Chlamydia muridarum* infected CD4^+^T cells C57BL/6 mice (*n* = 3) were challenged intravaginally with 5 × 10^4^ IFU Cm. Splenocytes were collected at day 12 *post* infection and CD4^+^T cells purified. Real time PCR was performed for murine **a.** ***FOXO-1***; and **b.** miR-182 and -183. Results are representative of 3 (FOXO-1) and 4 (miR-182 and-183) independent experiments. *P < 0.05 Student's *t* test compared to mock control infected mice. GAPDH or Snord 68 and 85 used as housekeeping gene or miRs, respectively. **c.** C57BL/6 mice (*n* = 5/group) were vaccinated intranasally with 500 IFU Cm or PBS (mock), rested for a month and subsequent intravaginal infection with 5 × 10^4^ IFU Cm or PBS (mock). Splenic CD4^+^T cells were purified at day 6 *post* infection and miR-182 and -183 expression was assessed. Results are representative of 2 independent experiments. *P* < 0.05 using ANOVA with Tukey-B *compared to mock vaccinated + mock infected mice. ^§^Cm vaccinated + Cm infected compared to Cm vaccinated + mock Infected. ^Ψ^Cm vaccinated + Cm infected compared to mock vaccinated + Cm Infected mice. **d.** Non-supervised hierarchical clustergram depicting miR-182 and -183 co-regulation in vaccinated and infected groups. **e.** ELISPOT revealed significant increase in antigen-specific IFN-γ /10^6^ splenocytes in Cm vaccinated + Cm infected compared to other groups at day 6 *post* Cm infection. *P* < 0.05 using ANOVA with Tukey-B *P* < 0.05*^,^**^,^*** compared to mock vaccinated + mock infected mice. ^§^Cm vaccinated + Cm infected compared to Cm vaccinated + mock infected control. **f.** Bacterial shedding; **g.** Percent mice positive for Cm in all groups (*n* = 5/group) was monitored and robust protection was observed in Cm vaccinated + Cm infected compared to mock vaccinated + Cm infected mice (*P* < 0.05 = Φ). ND = Not detected. Dotted line indicates below the level of detection.

Efficient Ag presentation by DC results in clonal proliferation of effector T-cell populations for generation of anti-Cm immunity [1, 26]. Using qRT-PCR, we measured expression of *FOXO-1*, a forkhead transcription factor expressed in resting T-cells and a suppressor of proliferation to functional T-cells [33]. We observed significant down-regulation of *FOXO-1* in splenic CD4^+^ T cells isolated at day 12 from Cm infected mice compared to mock infected animals (Figure 4a). We next, determined expression of miR-182 previously reported to regulate *FOXO-1* expression [34, 35]. MiR-182 was observed to be significantly up-regulated in day 12 CD4^+^ T cells from Cm infected mice compared to mock infected animals (Figure 4b) demonstrating an inverse correlation between miR-182 and *FOXO-1* expression in CD4^+^ T cells.

We further hypothesized that miR-182 regulation in Ag-specific CD4^+^ T cells (*i.e.,* the primary effector cells during the adaptive immune phase of a genital Cm infection [27]) might potentially be involved in protection against a subsequent genital Cm infection. Therefore, we vaccinated (intranasally) groups of mice with Cm and after resting, subsequently infected intravaginally with Cm. We observed significant up-regulation of miR-182 in day 6 splenic CD4^+^ T cells from Cm vaccinated + Cm infected mice compared to mock vaccinated + mock infected animals (Figure 4c). MiR-182 expression was also observed to be significantly up-regulated in Cm vaccinated + Cm infected mice compared to mock vaccinated + Cm infected mice or Cm vaccinated + mock infected animals (Figure 4c). Additionally miR-183 (a member of the miR-182 family [35]) was found to be significantly up-regulated in day 12 CD4+ T cells from Cm infected mice (Figure 4b), and in Cm vaccinated + Cm infected mice (Figure 4c) compared to control. Importantly, similar heat intensity profiles (light to dark) of a non-supervised hierarchical clustergram were indicative of probable co-regulation and expression of miR-182 and -183 in vaccinated mice; thereby, suggesting similar roles for both miRs in Ag-specific immune responses (Figure 4d). Expression of miR-182 correlated with protection against subsequent Cm infection at day 6 *post* infection as determined by production of Ag-specific IFN-γ ( determined by ELISPOT) (Figure 4e), and resolution of infection (Figure 4f) from all mice (Figure 4g) in Cm vaccinated + Cm infection compared to control groups. Taken together, regulation of miR-182 in Ag-specific CD4^+^ T cells from Cm infected and vaccinated mice suggests its contribution to generation of Ag-specific immune responses and protective immunity.

**Figure 5:**
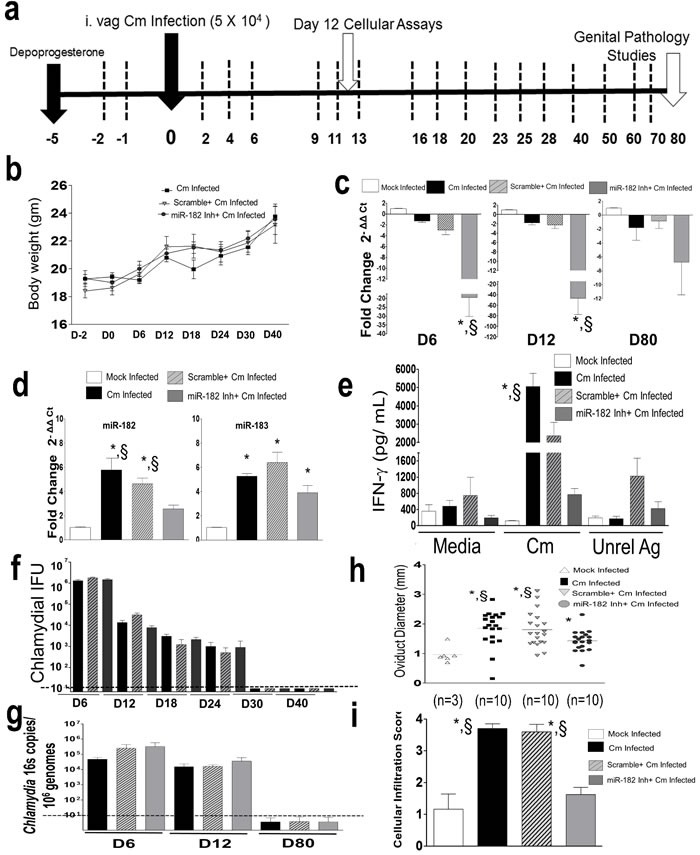
Murine miR-182 significantly regulates Ag-specific immune responses and disease pathology in *Chlamydia muridarum* infected mice C57BL/6 mice (*n* = 16/group) were challenged intravaginally with 5 × 10^4^ IFU Cm. **a.** Using a i.p depletion regimen, miR-182 was depleted using miR-182 inhibitor or controls (scramble treated, mock treated) at each of the time points indicated by dashed lines **b.** Monitoring of weight on indicated days revealed no significant changes due to treatment. *P* < 0.05 using ANOVA with repeated measures. Significant regulation in miR-182 expression in **c.** genital tract (down-regulation compared to mock) and **d.** splenic CD4^+^T cells (up-regulation compared to mock) collected at indicated time points (*n* = 3/ group/ time point) was observed. *P* < 0.05 using ANOVA with Tukey B Comparison Test in **c.**, * miR-182 inhibitor treated Cm infected mice compared to Cm infected mice; ^§^ miR-182 inhibitor treated Cm infected mice compared to scramble treated Cm infected mice and **d.** significant increase in miR-182 and miR-183 expression * compared to mock infected mice; ^§^ compared to miR-182 inhibitor treated Cm infected mice. **e.** Splenocytes (*n* = 3) were collected at day 12 *post* infection and IFN-γ was measured 72 h *post* co-culture. Results are representative of 5 independent experiments. *P* < 0.05 using ANOVA with Bonferroni's Multiple Comparison Test * Cm infected mice compared to mock infected mice. ^§^Cm infected mice compared to miR-182 inhibitor treated Cm infected mice. **f.** Bacterial shedding, **g.** tissue bacterial burden qPCR of *Chlamydia* 16s encoding gene (at indicated time points), **h.** oviduct pathology and **i.** total cellular infiltration were evaluated in all groups of mice. Each dot is representative of an oviduct. Results are representative of 2 independent experiments. Kruskall-Wallis test was used for comparing bacterial shedding profiles and histopathology. *P* < 0.05 * compared to mock infected mice. ^§^ compared to miR-182 inhibitor treated Cm infected mice. Dotted line indicates below the level of detection.

Given that miR-182 was up-regulated in Ag-specific CD4^+^ T cells from Cm infected and vaccinated mice, we determined the *in vivo* relevance of miR-182 in genital Cm infection. We used a depletion regimen for up to day 80 post infection (Figure 5a) which had no differential effect on weight change (Figure 5b), obvious health and wellness of mice and as expected, lead to significant reduction in miR-182 expression in genital tract (Figure 5c) or splenic CD4^+^ T cells (Figure 5d) isolated from miR-182 inhibitor treated mice following Cm infection compared to Cm infected mice at the assessed time points. We observed significant reduction in Ag-specific IFN-γ levels in splenocytes isolated from miR-182 inhibitor treated mice compared to Cm infected mice or scramble treated mice at day 12 *post* infection (Figure 5e). While comparable bacterial shedding profiles between mock-treated, scramble- or miR-182 inhibitor treated Cm infected mice over a 30 day *post* infection period using culture (Figure 5f) or 16s PCR (at indicated time points, namely days 6, 12 and 80 *post* infection, Figure 5g) were observed, we found significant reduction in oviduct pathology (Figure 5h) and total cellular infiltrates (Figure 5i) in miR-182 inhibitor treated mice compared to Cm infected mice or scramble treated mice. Mock infected and Cm infected mice with vehicle alone treatment displayed immune profiles, shedding and genital pathology comparable to mock infected and Cm infected mice respectively (data not shown). Additionally, despite miR-183 being co-regulated with miR-182 (Figure 4d), miR-182 inhibitor failed to significantly downregulate miR-183 expression in day 12 splenic CD4^+^ T cells thereby indicating the specificity of the depletion regime for miR-182. Importantly, while Ag-specific IFN-γ levels are critical for clearance of Cm from mice, it also leads to collateral damage to genital tissue architecture and development of upper genital pathology. Taken together our results (Figures 4-5) demonstrate that miR-182 upregulated in Ag-specific CD4^+^ T cells directly contributes to Ag-specific IFN-γ and Cm infected associated disease pathogenesis *in vivo*.

### Interferon-γ production in *C. muridarum* infected dendritic cells and CD4^+^ T cells is co-regulated by miRs-155 and -182

**Figure 6:**
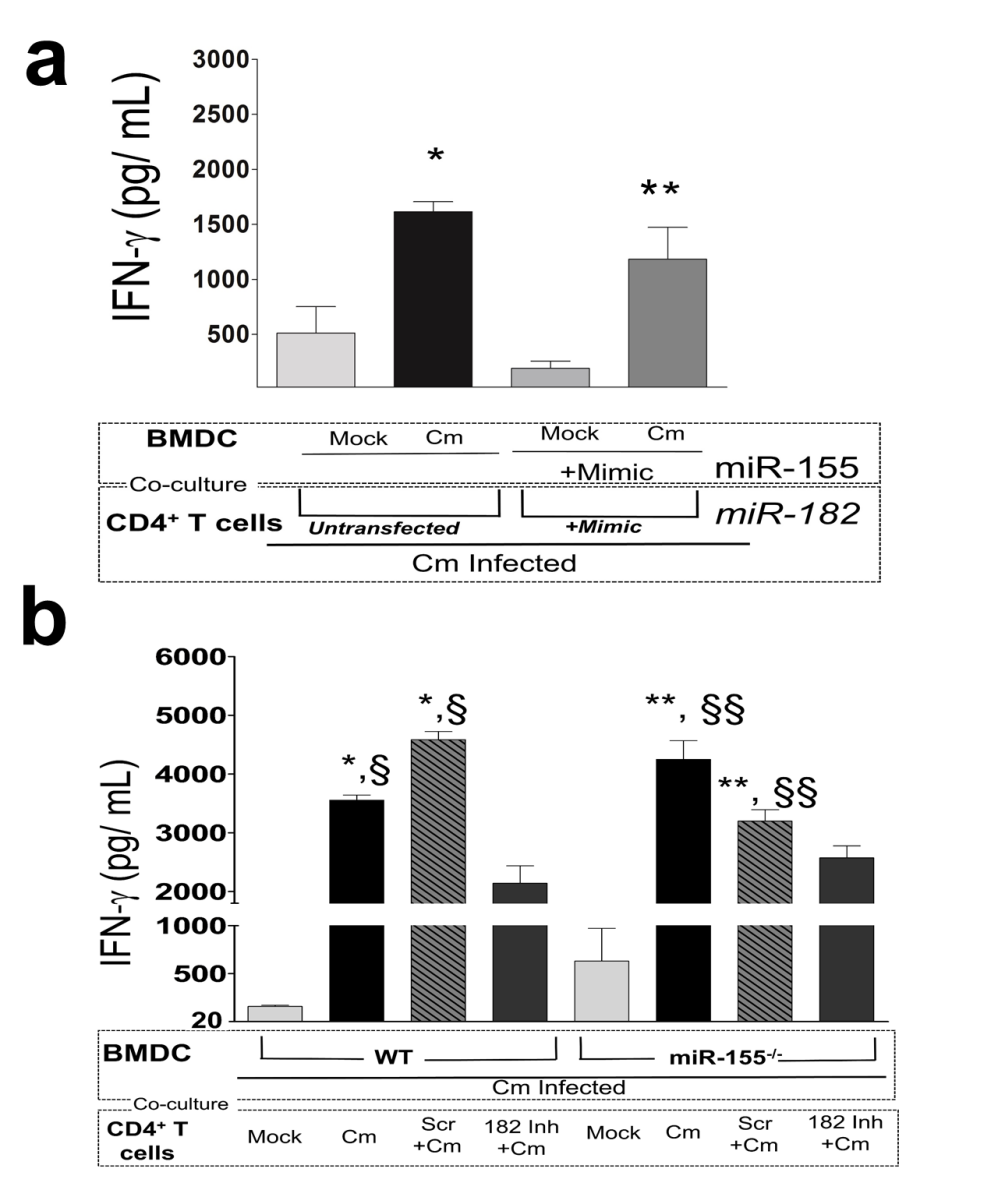
Interferon-γ production in *Chlamydia muridarum* infected dendritic and CD4^**+**^ T cells is co-regulated by miR-155 and -182 **a.** C57BL/6 mice (*n* = 5) were challenged intravaginally with 5 × 10^4^ IFU Cm. Splenocytes were collected at day 12 *post* infection and CD4^+^T cells purified. IFN-γ was measured 72 h *post* co-culture in miR-155 mimic or untransfected BMDC mock or Cm infected (MOI = 1) and at 24 h *post*-infection co-cultured with CD4^+^T cells. Co-cultured CD4^+^T cells were mock transfected or transfected with miR-182 mimic. *P* < 0.05 using ANOVA with Tukey-B. *Cm infected, untransfected BMDC compared to mock infected, untransfected BMDC. **miR-182 mimic transfected CD4^+^T cells co-cultured with miR-155 mimic Cm infected BMDC compared to miR-182 mimic transfected CD4^+^T cells co-cultured with miR-155 mimic, mock infected BMDC. **b.** C57BL/6 mice (*n* = 3) were challenged intravaginally with 5 × 10^4^ IFU Cm. Groups of mice were depleted for miR-182 using regimen described in Figure 5a. Splenocytes were collected at day 12 *post* infection and CD4^+^T cells purified. IFN-γ was measured 72 h *post* co-culture in miR-155^−/−^ or WT BMDC Cm infected (MOI = 1) and at 24 h *post*-infection co-cultured with CD4^+^T cells. *P* < 0.05 using ANOVA with Tukey-B. * compared to Cm infected WT BMDC co-cultured with CD4^+^T cells isolated from mock infected mice. ^§^ compared to Cm infected WT BMDC co-cultured with CD4^+^T cells isolated from miR-182 inhibitor treated Cm infected mice. ** compared to Cm infected miR-155^−/−^BMDC co-cultured with CD4^+^T cells isolated from mock infected mice. ^§§^ compared to Cm infected miR-155^−/−^BMDC co-cultured with CD4^+^T cells isolated from mock infected mice. Results are representative of 2 independent experiments. *P* < 0.05 using ANOVA with Bonferroni's Multiple Comparison Test.

Since IFN-γ is the key cytokine involved in adaptive immunity against Cm infection, and is produced by Ag-specific CD4^+^ T cells from Cm infected or vaccinated mice [27], we determined the contribution of miR-182 in generation of Ag-specific IFN-γ. As previously indicated, cf. Figure 3b, we observed significant production of IFN-γ in Cm infected, untransfected BMDC co-cultured with untransfected CD4^+^ T cells isolated at day 12 *post* infection from Cm infected mice (Figure 6a). Moreover, since miRs-155 and -182 were significantly up-regulated in BMDC (Figure 1) and CD4^+^ T cells (Figure 4) respectively, we also determined the effect of co-culturing miRs-155 and -182 treated cells. IFN-γ levels were observed to be significantly increased in Cm infected, miR-155 mimic transfected BMDC when co-cultured with miR-182 mimic transfected, CD4^+^ T cells isolated from Cm infected mice compared to mock infected BMDC (Figure 6a). This increased IFN-γ was comparable to untransfected co-cultures (Figure 6a).

In order to corroborate these findings, we utilized our *in vivo* depletion regimen (Figure 5a) and isolated splenic CD4^+^ T cells from mock treated, scramble treated and miR-182 inhibitor treated mice at day 12 post Cm infection. As shown in Figure 6b, Ag-specific IFN-γ levels were significantly reduced in Cm infected WT BMDC cocultured with CD4^+^ T cells from miR-182 inhibitor treated mice compared to Cm infected mice. Importantly, this significant difference in IFN-γ levels was ‘lost/ rescued in’ upon co-culture of Cm infected miR-155^−/−^ BMDC with CD4^+^ T cells from miR-182 inhibitor treated mice and was comparable to levels in Cm infected WT BMDC cocultured with CD4^+^ T cells from Cm infected mice (Figure 6a).

Overall, these data using mimics, inhibitors and *in vivo* depletion strategies strongly suggest that miRs 155 and -182 together compensate for the individual effect of each miR on production of IFN-γ and co-regulate the production of IFN-γ in co-cultures of Cm infected DC and CD4^+^ T cells (as shown summarized in Supplementary Table 2).

## DISCUSSION

There is growing evidence that miRs contribute to critical processes including immune cell development/function [13], reproductive biology [16, 36], and vaccination [37, 38]. We have previously reported the potential of miRs to regulate immunity in the genital tract of Cm infected mice from days 6 and 12 *post* infection [11]. These time points of analyses were chosen as respective representatives of initial and subsequent stages of host immunity and genital Cm infection in the well-established murine model described by several laboratories including ours [3, 4, 7-10]. Additionally, we have reported that miR-214, selected from our initial report [11], specifically regulates *ICAM-1* in the Cm infected genital tract, and contributes to causation of upper genital pathology in wild type and IL-17A deficient mice [18]. However, given that our initial reports [11] focused on the genital tract tissue and that the specific cell contributing to the miR responses was not investigated, we extended our hypothesis to investigate the role of immune cell specific miRs in the current study. This study provides insights on specific miRs from two key immune cell types involved in the interaction between innate and adaptive immunity *post* Cm infection. Although it is well established that following a genital infection with Cm, DC are the principle cell populations that professionally present Ag to CD4^+^ T cells to mediate protective immunity, and were hence the choice of cells for this initial study, the role of miRs in other immune cell populations requires future investigation [1, 7, 39, 40]. Importantly, this study underscores the growing importance of miRs as regulators/modulators of host processes in *Chlamydia* biology [19, 21, 23, 41-43] at its naturally infecting sites including the lung, eye and the female reproductive tract-where the role of miRs in immune regulation in other models have been well documented [15, 44, 45]. Additionally, it extends our initial report on the role of miRs in modulating host immunity in the genital tract of Cm infected mice [11].

Identification of miRs in cultured DC infected with Cm revealed only 12/88 miRs probed being regulated compared to mock infected controls (Figure 1, Supplementary Table 1). Of these, the contribution of miR-155 was investigated here for its well- established role in modulating host immunity [30, 31]. Using inhibitors and mimics of miR-155, we established its contribution in activation of DC by regulation of MHC-II expression in CD11c^+^ BMDC (Figure 2). These observations are in agreement with the findings of Dunand-Sauthier *et al*., on the regulation of MHC-II in miR-155 deficient mice [32]. Additionally, up-regulation of miR-155 in C57BL6 mice infected with Cm mutant strains as reported by Yeruva *et al*., and the recent report on the relationship of miR-155 and -184 in ocular Ct infection associated inflammation are indicative of the intrinsic contribution/ role of miR-155 in Ct infections [23, 46]. Importantly, we found that co-culture of miR-155 inhibitor and mimic transfected BMDC or miR-155^−/−^ BMDC with CD4^+^ T cells isolated at day 12 *post* infection from Cm infected mice resulted in significant modulation of Ag-specific IFN-γ production (Figure 3b). We co-cultured BMDC with Ag-specific CD4^+^ T cells isolated specifically from day 12 *post* infection from Cm or mock infected mice as it is well established that the CD4^+^ T cells are (1), the primary source of Ag-specific IFN-γ production [27]; (2), CD4^+^ T cells are the most important effector cell population that have the potential to provide protective responses against a subsequent infection [9] and; (3), adaptive immune responses are initiated around days 12-15 *post* infection in a well characterized 27-33 day murine genital infection model [7, 10]. Increased levels of IFN-γ production correlated with significant up-regulated expression of transcription factor *Tbx21* indicating clonal expansion of Th1 cells (data not shown). Interestingly, regulation of MHC-II by miR-155 inhibitor (Figure 2), and IFN-γ production when these treated cells were co-cultured with Ag-specific CD4^+^ T cells (Figure 3) were not observed as co-related. However, given that Ag-priming and cognate binding is dependent on several factors including MHC-II levels, we speculate that the probable mechanisms in the T cell component may exert its effect in contributing to IFN-γ production. Additionally investigations into regulation of activation of APC (*i.e.,* MHC-II) by miR-155 *via* pathways involving molecules such as c-Fos, SHIP1, SOCS-1 and cytokines, IL-12 and IL-6, are warranted for a better understanding of Ag-specific IFN-γ production in Ct infections [32, 47, 48].

Our results also demonstrate the down-regulation of transcription factor *FOXO-1*, and up-regulation of its controlling microRNA, *i.e*., miR-182 [35], in CD4^+^ T cells isolated at day 12 *post* infection from Cm infected mice compared to mock infected animals (Figure 4). Importantly, miR-182 and its family member, miR-183 were significantly co-regulated in CD4^+^ T cells isolated from vaccinated mice protected at day 6 after a subsequent intravaginal Cm infection (Figure 4c, 4d). Collectively, these results suggest miR-182 may potentially be critically involved in initiation of adaptive immunity following Cm infection (isolated at day 12 *post* infection) and may also be involved in vaccine mediated protection (isolated at day 6 *post* infection in vaccinated mice). We have previously reported that miRs 182 and -183 are significantly modulated in the Cm infected genital tract, and are up-regulated in the lower genital tract of Cm infected CD4^−/−^ mice compared to the wild type [11]. Taken together, data from both studies suggest that the miR-182 family is involved in genital Cm infection, and has cell- and tissue-specific function in infected mice. Additionally, altered immune responses in miR-182 deficient mice [49], [50] highlights the importance of further investigating the role of Cm-specific immune responses in this genetically deficient background. We thus employed a miR-182 depletion strategy to demonstrate the direct contribution of miR-182 to Ag-specific IFN-γ production. To this end, miR-182 inhibitors were formulated in polymeric, PEI-based nanoparticles for *in vivo* delivery [51, 52]. In accordance with our previous findings [11], we observed a significant reduction in miR-182 expression in genital tracts (Figure 5c) or in CD4^+^ T cells (Figure 5d) isolated from miR-182 inhibitor treated mice compared to control-treated or mock treated mice *post* Cm infection. The significant reduction in Ag-specific IFN-γ was associated with significant decrease in development of upper genital pathology in miR-182 inhibitor treated mice compared to Cm infected mice (Figure 5e). Additionally, comparable levels of bacterial shedding in miR-182 inhibitor treated and control groups of mice is in agreement with our previous report on the down-regulation of miR-182 in murine genital tracts following Cm infection or colonization within infected hosts [11]. Given that miR-182 is down-regulated in Cm infected mice, the *in vivo* depletion regimen (Figure 5a) resulted in an environment that would aid Cm colonization in the genital tract [11]. Also, while the level of Ag-specific IFN-γ production (Figure 5e) was significantly reduced in miR-182 depleted mice, the lack of a significant increase in bacterial shedding (Figure 5f, 5g) can be compared to previous findings on early to mid stage shedding in WT and IFN-γ signaling deficient mice [53, 54], the contribution of other molecules/ immune cells altered *in vivo* to this end cannot be ruled out. Significant reduction in upper genital pathology and total cellular infiltrates in miR-182 inhibitor treated mice, compared to mock or scramble treated mice, following Cm infection (Figure 5h, 5i) were in accordance with previous reports on the reduced inflammatory cellular infiltrates contributing to reduced genital pathology [53, 55-58]. Additionally, a recent study by Ichiyama *et al*., establishes the role of miR-182-183-96 cluster in driving Th_17_ pathogenicity *via* inhibition of *FOXO-1* [59, 60]. The *FOXO-1*-miR cluster regulatory axis in this report adds to (1), our findings (Figure 4) and (2), the understanding of the mechanistic contribution of IL-17A (a member of the Th17 family) in chlamydial pathogenesis. We [18] and others [55] have reported that *i. vag* Cm infected IL-17A deficient mice compared to WT mice display significantly abrogated bacterial shedding kinetics and genital pathology. Additional studies using lineage specific mice will delineate the contribution of miR-182 cluster regulation of Th_1_ and Th_17_ specific host immunity and its relative effect on development of upper genital pathology following Cm infection [59].

We established a role for miR-182 in regulating IFN-γ production in an Ag-specific manner (Figure 6). A significant increase in IFN-γ production was observed when miR-155 mimic transfected BMDC was co-cultured with miR-182 mimic transfected CD4^+^ T cells isolated from Cm infected mice compared to mock infected BMDC (Figure 6a). These levels were comparable to un-transfected conditions. This ‘co-culture’ combination of using miR mimics for both cell types provided a surrogate model to mimic the up-regulation of miRs 155 and -182 in Cm infected cells. Additionally, using a miR-155^−/−^BMDC: miR-182 depleted CD4^+^ T cell co-culture (Figure 6a), we corroborated the interaction of the 2 miRs and the co-regulatory effect on IFN-γ production (as summarized in Supplementary Table 2). Importantly, these findings were in agreement with *in vivo* observations on DC-CD4^+^ T cell interactions which lead to significantly increased IFN-γ production and protective responses upon genital Cm infection [26, 27].

Overall, this study provides novel information on regulation of ‘anti-Cm’ immunity by cell type specific miRs. However, the current study lacks data on specific targets of miRs 155 and -182 in BMDC or CD4^+^ T cells, respectively. Given that (1), both miRs-155 and -182 were up-regulated following Cm infection (Figures 1, 4); (2), co-culture of unmanipulated CD4^+^ T cells with over-expressed miR-155 DCs (Figure 3), or unmanipulated DCs with over-expressed miR-182 CD4^+^ T cells (data not shown) respectively, lead to reduction in Ag-specific IFN-γ; (3), miR-182 was observed to be up-regulated in vaccinated mice protected against a subsequent Cm infection where protection may be mediated by production of Ag-specific IFN-γ (Figure 4) and (4), miRs act by damping the target gene/ gene product, that additional investigation is warranted on IFN-γ signaling specific genes and regulators whose function is altered by these two miRs and result in Ag-specific IFN-γ (Figure 6) required for controlling Cm infection. Importantly, the availability of miR-155 and -182 deficient mice [32, 61], and the use of transfection strategies to determine miR-mRNA targets [32] upon Cm infection will be key and aid in determining the specific targets of these miRs that regulate downstream IFN-γ production.

This study establishes the specific contribution of two miRs in generation of anti-Ct immune responses and provides new information that can be beneficial for the rationale design of an effective anti-Ct vaccine. Moreover, given that (1), Ct is the leading cause of STIs globally [5], (2) a key mucosal pathogen in the female genital tract [62], and that (3), co-infections and the urogenital microbiome may be affected by and/ or influence Ct induced host immune responses [63-65], investigations on the role of miRs in regulating inherent host functions at the genital microenvironment are timely and essential for formulating paradigm shifting concepts.

## MATERIALS AND METHODS

### *Chlamydia muridarum* stocks

Seed stocks of Cm were propagated in HeLa 229 cells. At 24 h post infection, HeLa cells were mechanically disrupted, and following high speed centrifugation, bacterial pellets were purified on Renografin gradient as previously described [27].

### Mice

All procedures were carried out in compliance with Institutional Animal Care and Use Committee (IACUC) guidelines. Female, 4-6 week old wild type C57BL/6 (WT) mice and miR-155^−/−^ were purchased from Jackson Laboratory.

### Intravaginal challenge

In order to render the mice anestrous and more receptive to the genital infection, a single subcutaneous injection containing 2.5 mg Depo progesterone (Depo-Provera; Pharmacia & Upjohn Co., NY, USA) was administered five days prior to infection. Mice were intravaginally infected with purified Cm diluted in 15 μL sucrose/phosphate/glutamate (SPG) buffer at a final inoculating dose of 5 × 10^4^ IFU [27]. For miR-182 depletion studies, using a timeline (Figure 5a), mice were treated intraperitoneally (i.p) with 10 mg / mouse/ time point scramble- and miR-182 inhibitors. Bacterial shedding and upper genital pathology was assessed as described previously [27]. At day 80 *post* infection, genital tracts were removed from all groups of mice, fixed in 10% neutral formalin, and embedded into paraffin blocks. Serial horizontal sections (5 μm) were prepared and every tenth section (∼8-10 sections per tissue) was stained using hematoxylin and eosin (H&E). Stained sections were visualized using a Zeiss Axioskop 2 Plus research microscope and images were acquired using an Axiocam digital camera (Zeiss, Thornwood, NY). Representative sections stained with H&E were scored in a blinded fashion by a trained pathologist using a scoring scheme as described previously [66]. Results were expressed as mean ± SEM of scores from all animals in a group (*n* = 10). From a subset of mice (*n* = 3)/ treatment group genital tract (days 6, 12, 80) and splenic CD4^+^ T cells (day 12) were collected and stored in −80° C till further use. Genital tissue was ground using mortar and pestle and the crushed tissue was divided into 2 portions for isolating RNA (for miR PCR) and DNA (16s encoding gene PCR) respectively. Expression of miR-182 and -183 using miR-specific RT-PCR was performed as described below and bacterial burdens (days 6, 12, 80 for *Chlamydia* 16s encoding gene PCR from genital tract) were determined using primers and PCR parameters as described by Wooters *et al* [67].

### Vaccination studies

For vaccination studies, mice were intranasally immunized with 500 IFU live Cm and rested for a month. Following intravaginal infection with 5 × 10^4^ IFU Cm, mice were swabbed at days 2, 4, and 6 for cervical-vaginal bacterial shedding. At day 6 *post* Cm infection, mice were euthanized for isolating splenocytes.

### Cell cultures

#### Bone marrow derived dendritic cells (BMDC)

Female C57BL/6 mice (*n* = 3-5/ experiment) were euthanized and bone marrow was collected from the tibia and femur under aseptic conditions. BMDC were prepared as described previously with slight modifications [26]. At day 7 of culture, all cells were pooled and enriched using CD11c positive selection kit (Stem Cell Technologies, Vancouver). Following enrichment (purity > 85% by flow cytometry), BMDC were cultured in 96 well plates and treated with miR-155 mimic and/or inhibitor as described below.

#### CD4^+^ T-cells

At day 12 post infection, mock or Cm infected mice were euthanized and spleens isolated. Single cells were prepared as described previously [26]. CD4^+^ T-cells were purified (purity > 85-90% by flow cytometry) using a CD4 enrichment protocol per manufacturer's recommendation (StemCell). CD4+ T-cells were manipulated with miR-182 mimic prior to co-culture with treated BMDC.

#### Co-culture

At 24 h *post* Cm infection in treated BMDC, CD4^+^ T-cells were over-layed at a BMDC: CD4 ratio of 1:2.5 (for miR-155 manipulation experiments), and 1:2 (for miR-155 and -182 treatment experiments). Co-cultured cells were incubated at 37°C for 72 h. After 72 h, cells were centrifuged at 1200 rpm at 4°C for 10 min, and the supernatant was collected and stored at −80 °C for ELISA.

#### MicroRNA inhibitors formulation for *in vivo* depletion studies

For miR-inhibitor delivery, polymeric nanoparticles based on polyethylenimine (PEI, vehicle) were employed. More specifically, PEI F25-LMW / inhibitor complexes was prepared as previously described for siRNA [51]. In brief, 5 mg of the inhibitor mmu-miR-182-5p or the scrambled negative control oligonucleotide (IDT, Coralville, IA) were dissolved in 5 ml HN buffer (10 mM HEPES, 150 mM NaCl, pH 7.4), yielding a 400 nM stock solution. For complexation, 110 μl of this stock solution were diluted in 761 μl HN buffer. In parallel, 825 μg PEI F25-LMW [52] was diluted to a final volume of 780 μl in HN buffer. After incubation for 10 min, the PEI solution was added to the inhibitor solution, vortexed and incubated for 30 min at room temperature. For the complexation of smaller amounts, 40 μg oligonucleotide in 242 μl HN buffer was complexed with 300 μg PEI F25-LMW in 320 μl HN buffer in the same manner. Complexes were stored in −80°C prior to use.

#### *In vitro* transfection of microRNA mimics and inhibitors

BMDC and CD4^+^ T-cells were seeded at a density of 2 × 10^5^ or 5 × 10^5^ respectively, per well. MiR-155 or -182 agomiR (MiScript miRNA Mimics, Qiagen, Valencia, CA), and antagomiR (MiScript miRNA Inhibitor) were transfected using Attractene as transfection reagent at a concentration of 20 μM by fast forward transfection per manufacturer's recommendation (Qiagen). Transfection was carried out for 24 h (BMDC), and 18 h (CD4^+^ T-cells) followed by Cm infection (BMDC) or co-culture with infected BMDC (CD4^+^ T-cells). Prior to infection or co-culture, efficiency of transfection or *in vivo* depletion and cell viability was determined by Real Time PCR and MTS assay (Promega, Madison, WI), respectively, (data not shown).

#### RNA extraction and quantitative reverse transcriptase (qRT)-PCR

Total RNA was extracted from BMDC and splenic CD4^+^ T-cells at 24 h *post* infection or 12 days *post* infection, respectively. For vaccination studies, splenic CD4^+^ T-cells were isolated 6 days *post* infection. RNA was obtained using a miRNeasy RNA extraction Kit (Qiagen) according to the manufacturer's instructions. Total RNA was assessed using a Nanodrop Spectrophotometer (ThermoScientific, Asheville, NC). RNA samples exhibiting A_260/280_ 2.0 and A_260/230_ 1.8 were converted to cDNA using a RT2 First Strand cDNA Kit (Qiagen) according to the manufacturer's instructions. RNA (1 μg) was used in all miR PCR amplifications, and were performed using custom designed miScript miR-155, -182, and -183 primers (Qiagen) according to the manufacturer's instructions. For *FOXO-1, Tbx21, FoxP3, GATA3*, and *RoRγ* expression, RNA (1 μg) was converted to cDNA using an iScript^™^ cDNA Synthesis Kit followed by real time PCR using the SsoAdvanced^™^ Universal SYBR®Green Supermix (Bio-Rad, Hercule, CA) with gene specific primer pairs (PrimePCR^™^ SYBR® Green Assay, BioRad, UniqueAssay ID: FOXO-1 qMmuCID0016391) on a CFX 96 instrument (Bio-Rad). All miR expression analyses were normalized to housekeeping miRs RNU6-2_1 and SNORD68 expression values (for BMDC experiments) and SNORD68 and 85 (for CD4^+^ T-cell experiments). All gene expression analyses were normalized to housekeeping genes GAPDH. Data are represented as fold-change relative to mock control as indicated in the Results and Figure legends using the comparative cycle threshold method [11].

#### Flow cytometry

Cells were stained with fluorochrome-conjugated CD-11c, and MHC-II labeled antibodies (Biolegend, San Diego, CA), and FACS analysis was performed using a LSR II instrument (BD Biosciences) [27].

#### ELISA

Supernatants from ‘co-culture’ wells were analyzed for IFN-γ production using BD OptELISA kits (BD Pharmingen, NJ, USA) as previously described [26]. Cells were stimulated with media, BSA, live Cm (or UV-Cm, mentioned in figure legend) and non related antigens (*Myelin Oligodendrocyte Glycoprotein MOG*_35-55_).

#### ELISPOT

At day 6 *post* Cm infection, splenocytes were isolated from all groups of mice in vaccination studies, and single cell suspensions made as previously described [26]. IFN-γ ELISPOT was performed on 96-well MultiScreen HTS filtration plates (Millipore), and results captured on an ImmunoSpot Series 3 analyzer (Cellular Technology, Ltd., OH) as *per* specifications [68].

#### Statistical analyses

MiR analysis was performed using RT^2^ Profiler PCR Array Data Analysis (version 3.5, Qiagen). All experimental results were calculated as the mean ± SE of 2-5 independent experiments (*n* = 3-5) indicated in respective figure legends. Student's *t*-test was used for comparison between two groups. ANOVA with *post-hoc* multiple comparison test was used for multiple groups. Kruskall-Wallis test was used for comparing bacterial shedding profiles. Differences were considered statistically significant if P values were < 0.05. All statistical analyses were conducted using the GraphPad Prism 5 software package (La Jolla, CA).

## SUPPLEMENTARY MATERIAL TABLES


