# Supplementary material for: Antigen specific immune response in Chlamydia muridarum genital infection is dependent on murine microRNAs-155 and -182
Source: Oncotarget. 2016 Aug 20;7(40):64726–42. doi: 10.18632/oncotarget.11461 (PMC5323111; doi:10.18632/oncotarget.11461)
Supplement: Supplementary file 1 [file oncotarget-07-64726-s001.pdf]

Antigen specific immune response in *Chlamydia muridarum* genital infection is dependent on murine microRNAs-155 and -182

Supplementary Material

Supplementary Table 1. Regulation of murine microRNAs in *Chlamydia muridarum* infected dendritic cell cultures

MicroRNAs	Fold Change (Arbitrary Units)	95% Confidence Interval	P value
Down-regulated compared to mock infected			
miR-149	-2.5	0.34, 0.46	0.001
miR-223	-2.01	0.30, 0.70	0.04
miR-26a	-2.23	0.21, 0.68	0.03
Up-regulated compared to mock infected			
miR-155	2.98	1.05, 4.91	0.04
miR-214	3.20	1.06, 5.36	0.002
miR-34a	2.40	1.46, 3.36	0.016
miR-34c	3.29	1.37, 5.22	0.007
miR-363	2.78	0.82, 4.75	0.01
miR-451	2.63	1.38, 3.90	0.03
miR-187	2.10	1.09, 3.11	0.05
miR-298	2.13	0.32, 3.95	0.18
miR-383	2.05	0.14, 3.98	0.19

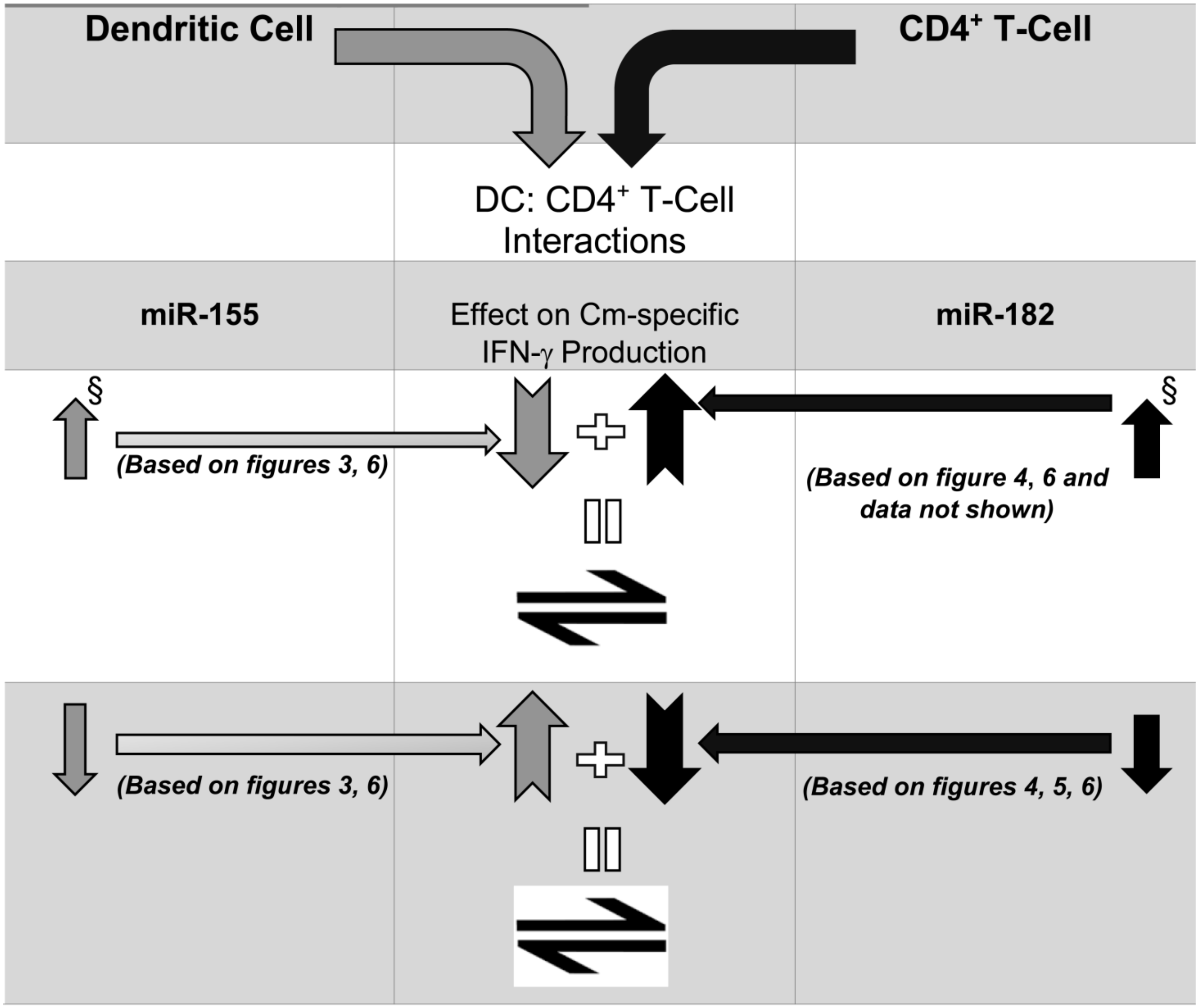

Supplementary Table 2. Interferon-γ production in *Chlamydia muridarum* infection is co-regulated by murine microRNAs -155 and -182.

Key

IFN-γ levels:

IFN-γ levels statistically greater or reduced compared to levels in unmanipulated wild type Cm infected cells.

Resultant IFN-γ levels statistically comparable to coculture with unmanipulated wild type Cm infected cells (representative of murine *in vivo* Cm intravaginal infection model). (ANOVA with *post hoc* tests, based on figure 6)

MiRs:

§ Found naturally upregulated following Cm infection in respective cell types.

⇧ ⬆ Upregulation of miR-155 or miR-182 compared to mock conditions was experimentally achieved by using mimics of respective miRs.

⇩ ⬇ Downregulation of miR-155 or miR-182 compared to mock conditions was achieved experimentally by using inhibitors, genetic mutants or depletion regimes for respective miRs.
